# Identification and characterization of the COPII vesicle‐forming GTPase Sar1 in *Chlamydomonas*

**DOI:** 10.1002/pld3.614

**Published:** 2024-06-16

**Authors:** Kin Pan Chung, Daniel Frieboese, Florent Waltz, Benjamin D. Engel, Ralph Bock

**Affiliations:** ^1^ Max‐Planck‐Institut für Molekulare Pflanzenphysiologie Potsdam Germany; ^2^ Biozentrum University of Basel Basel Switzerland

**Keywords:** *Chlamydomonas reinhardtii*, COPII vesicles, ER‐to‐Golgi protein trafficking, protein secretion, Sar1

## Abstract

Eukaryotic cells are highly compartmentalized, requiring elaborate transport mechanisms to facilitate the movement of proteins between membrane‐bound compartments. Most proteins synthesized in the endoplasmic reticulum (ER) are transported to the Golgi apparatus through COPII‐mediated vesicular trafficking. Sar1, a small GTPase that facilitates the formation of COPII vesicles, plays a critical role in the early steps of this protein secretory pathway. Sar1 was characterized in yeast, animals and plants, but no Sar1 homolog has been identified and functionally analyzed in algae. Here we identified a putative Sar1 homolog (CrSar1) in the model green alga *Chlamydomonas reinhardtii* through amino acid sequence similarity. We employed site‐directed mutagenesis to generate a dominant‐negative mutant of CrSar1 (CrSar1DN). Using protein secretion assays, we demonstrate the inhibitory effect of CrSar1DN on protein secretion. However, different from previously studied organisms, ectopic expression of CrSar1DN did not result in collapse of the ER‐Golgi interface in *Chlamydomonas*. Nonetheless, our data suggest a largely conserved role of CrSar1 in the ER‐to‐Golgi protein secretory pathway in green algae.

## 1 INTRODUCTION

Protein secretion represents a crucial biological process with significant implications in both fundamental research and recombinant protein production in biotechnology. In microalgae, protein secretion is not only pivotal for numerous physiological functions, including responses to nutrient deprivation (Lien & Knutsen, 1973; Loppes & Matagne, 1973; Schreiner et al., 1975), stress response (Coleman & Grossman, 1984; Kimpel et al., 1983; Moroney & Tolbert, 1985; Spalding et al., 1983; Tsuzuki, 1983), and sexual reproduction (Claes, 1971; Schlösser, 1976), but also holds substantial potential for the development of efficient platforms for the production of recombinant proteins (Einhaus et al., 2023; Ramos‐Martinez et al., 2017).

The protein secretory pathway is generally conserved across eukaryotes (More et al., 2020). Proteins synthesized in the endoplasmic reticulum (ER) are transported through various subcellular compartments including the Golgi apparatus and the *trans*‐Golgi network, eventually reaching the plasma membrane, and can then be secreted into the extracellular space (Aniento et al., 2022; Matheson et al., 2006; Stefano et al., 2014; Vitale & Denecke, 1999; Wang et al., 2018). This process is facilitated by vesicular trafficking, which transports proteins between the compartments involved (Barlowe & Miller, 2013; Hawes et al., 1999; Rojo & Denecke, 2008).

A prominent example of vesicular trafficking is the coat protein complex II (COPII), mediating the transport of proteins from the ER to the Golgi in the early secretory pathway (Raote et al., 2023). Extensive research in yeast, animals and plants has revealed the core components of the COPII machinery (Brandizzi, 2018; D'Arcangelo et al., 2013; Li et al., 2023; Peotter et al., 2019; Venditti et al., 2014; Zanetti et al., 2011). Central to the COPII machinery is the small GTPase Sar1, known for its crucial role in facilitating the formation of COPII vesicles (Antonny et al., 2001; Bi et al., 2002; Chung et al., 2016; Li et al., 2021; Yoshihisa et al., 1993).

Recent studies have expanded our knowledge of Sar1 and its role in COPII‐mediated protein trafficking. While Sar1 has been traditionally viewed as critical for initiating COPII vesicle formation, a recent study has proposed alternative roles and mechanisms for Sar1 in mammals (Kasberg et al., 2023). Research on *Arabidopsis thaliana* Sar1 homologs has revealed distinct roles of plant Sar1 isoforms that exhibit differences in localization, membrane association, and effects on ER protein export (Hanton et al., 2008; Liang et al., 2020, 2023; Zeng et al., 2015). These findings highlight the complexity and the multifarious functions of Sar1 isoforms in the secretory pathway.

To better understand the early protein secretory pathway in *Chlamydomonas*, we aimed to identify the *Chlamydomonas* Sar1 (CrSar1) and examine its role in ER‐to‐Golgi protein trafficking. Our findings indicate that Sar1 is highly conserved in algae and seed plants. By performing site‐directed mutagenesis of a critical amino acid residue in the GTP‐binding protein domain, we generated a dominant‐negative (DN) mutant of CrSar1 (hereafter, CrSar1DN) that impeded effective protein secretion. Intriguingly, protein secretion was not completely abolished, and the ultrastructure of the ER‐Golgi interface remained largely intact in the *Chlamydomonas* strain expressing the CrSar1DN protein. Taken together, our work has identified and characterized the *Chlamydomonas* Sar1 homolog and revealed its conserved role in protein trafficking.

## 2 RESULTS

### 2.1 Identification of the small GTPase Sar1 in *Chlamydomonas reinhardtii*

**FIGURE 1:**
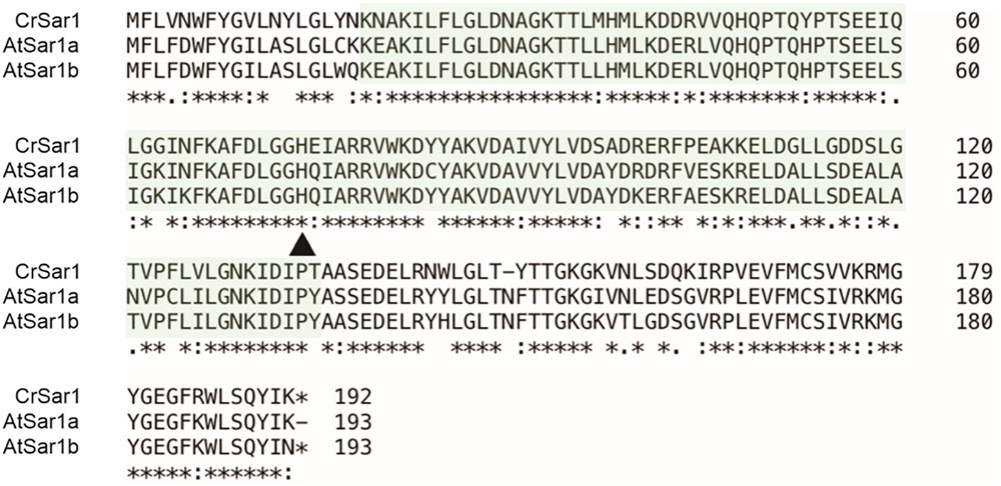
*Chlamydomonas* and *Arabidopsis* Sar1 proteins are highly conserved. Amino acid sequence alignment of the candidate Sar1 protein from *Chlamydomonas reinhardtii* (CrSar1) and the two *Arabidopsis thaliana* Sar1 proteins (AtSar1a and AtSar1b). The sequences were retrieved from the platform Phytozome 13 (https://phytozome‐next.jgi.doe.gov/). CrSar1 (encoded by Cre11.g468300) shares a high level of sequence similarity to AtSar1a (At1g09180, 74.61% identity) and AtSar1b (At1g56330, 76.04% of identity). The black arrowhead indicates the conserved histidine at position 74 [H74] that is involved in catalysis. The sequence shaded in green denotes the small GTP‐binding protein domain. The dominant‐negative mutant form of Sar1 (CrSar1DN) was created by a histidine‐to‐leucine substitution at position 74 [H74L].

To identify putative Sar1 homologs in *Chlamydomonas*, we performed an amino acid sequence‐based search using the basic local alignment search tool (https://blast.ncbi.nlm.nih.gov/Blast.cgi). Two characterized *A. thaliana* Sar1 homologs (Hanton et al., 2008), AtSar1a and AtSar1b, were used as query sequences for the search. We identified a single promising candidate, preliminarily termed CrSar1, that shares a high level of amino acid sequence identity with both AtSar1a (74.61% identity) and AtSar1b (76.04% identity; Figure 1). Notably, the sequence alignment data revealed conservation of the histidine residue known to be important for Sar1 GTP hydrolysis activity (Yorimitsu et al., 2014) (at position 74 of CrSar1; Figure 1). In addition, CrSar1 possesses the small GTP‐binding protein domain (Figure 1), suggesting its function as a small GTPase.

### 2.2 CrSar1 localizes in the vicinity of the ER

**FIGURE 2:**
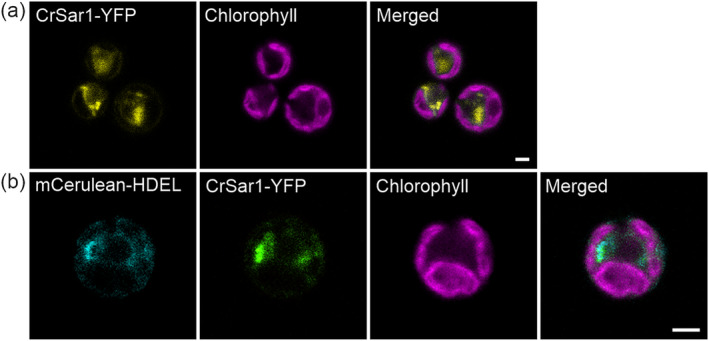
CrSar1‐YFP‐containing punctate structures localize in the vicinity of the endoplasmic reticulum (ER). Confocal microscopic images of transgenic *Chlamydomonas* strains (a) expressing CrSar1‐YFP and (b) co‐expressing the ER marker mCerulean‐HDEL and CrSar1‐YFP. Chlorophyll fluorescence is shown in magenta. Note that CrSar1‐YFP shows a diffused cytosolic pattern and a punctate pattern in proximity to the ER. Scale bars, 2 μm.

To investigate the potential role of CrSar1 in the early protein secretory pathway, we first wanted to determine its subcellular localization by confocal microscopy. To this end, we cloned *CrSar1* into an expression vector, generating a C‐terminal fusion to the yellow fluorescent protein (YFP; Figure S1a). By expressing CrSar1‐YFP in the *Chlamydomonas* expression strain UVM11 (Barahimipour et al., 2016; Neupert et al., 2009), we observed a cytosolic accumulation pattern of the YFP signal, with punctate structures distributed in the cytosol (Figure 2a). In *Arabidopsis* and tobacco, the Sar1‐containing punctate structures are closely associated with the ER, representing putative COPII assembly and ER exit sites (ERES) (McGinness et al., 2022; Takagi et al., 2020; Zeng et al., 2015). To further examine the CrSar1‐containing punctate structures in the alga, we next co‐expressed CrSar1‐YFP with the ER marker mCerulean‐HDEL (Rasala et al., 2014). Analysis of the generated transgenic strains by confocal laser‐scanning microscopy revealed that the CrSar1‐containing punctate structures largely co‐localize with the mCerulean‐HDEL reporter (Figure 2b), indicating a close association of CrSar1‐YFP with the ER.

### 2.3 Generation of a dominant‐negative mutant form of CrSar1

In *Arabidopsis*, it has been shown that a single amino acid substitution (histidine to leucine) at position 74 of AtSar1 creates a dominant‐negative mutant form of Sar1, by generating a catalytically inactive, so‐called GTP‐restricted form, Sar1DN (Takeuchi et al., 2001; Zeng et al., 2015). The ectopic expression of Sar1DN inhibits COPII vesicle formation, thereby impairing ER‐to‐Golgi protein trafficking. Consequently, Sar1DN represents a powerful tool for studying the early protein secretory pathway in plants (Osterrieder et al., 2010).

**FIGURE 3:**
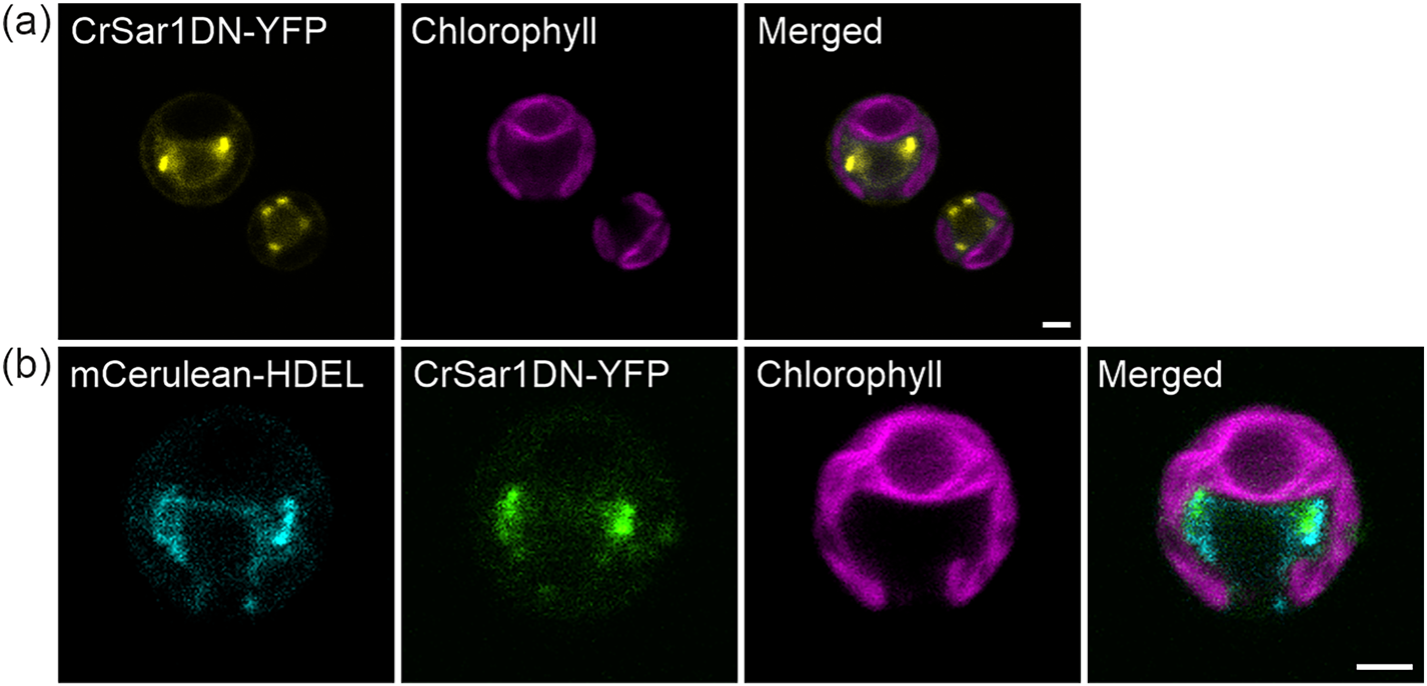
CrSar1DN‐YFP localizes to punctate structures in the vicinity of the endoplasmic reticulum (ER). Confocal microscopic images of transgenic *Chlamydomonas* strains (a) expressing CrSar1DN‐YFP, and (b) co‐expressing the ER marker mCerulean‐HDEL and CrSar1DN‐YFP. Chlorophyll fluorescence is shown in magenta. CrSar1DN‐YFP shows a diffuse cytosolic pattern and a pronounced punctate pattern proximal to the ER, similar to CrSar1‐YFP. Scale bars, 2 μm.

Because CrSar1 possesses the conserved histidine at position 74, we decided to explore the potential of creating a dominant‐negative, GTP‐restricted form of CrSar1 (CrSar1DN). By performing site‐directed mutagenesis, we introduced a histidine‐to‐leucine substitution at position 74 (H74L) into the *CrSar1* gene. To further characterize the CrSar1DN protein variant, we also subcloned the *CrSar1DN* into our expression vector for generating C‐terminal protein fusions to YFP (Figure S1b), to be able to study its subcellular localization. CrSar1DN‐YFP showed a similar localization pattern as CrSar1‐YFP, in that the protein largely accumulated in scattered punctuate structures within the cytosol (Figure 3a). Co‐expression of CrSar1DN‐YFP with the ER marker mCerulean‐HDEL revealed that the CrSar1DN‐containing punctate structures are present at the periphery of the ER (Figure 3b).

Taken together, our subcellular localization studies suggested that both CrSar1‐YFP and CrSar1DN‐YFP accumulate in punctuate structures that are closely associated with the ER, suggesting that they may be recruited to ERES to facilitate vesicle budding and protein export.

### 2.4 Ectopic expression of CrSar1DN‐YFP affects protein secretion

Having shown that the CrSar1DN‐containing punctate structures localize in the vicinity of the ER, we set out to investigate the impact of the ectopic expression of CrSar1DN‐YFP on protein secretion. To this end, we designed an assay that allows the evaluation of protein secretion efficiency using the protein cargo SS‐mCherry‐SP_10_.

SS‐mCherry‐SP_10_ represents a fusion protein: The signal sequence (SS) from the metalloprotease gametolysin (Ramos‐Martinez et al., 2017) and the synthetic glycomodules (SP_10_) (Ramos‐Martinez et al., 2017) were fused to the N‐terminus and the C‐terminus, respectively, of the fluorescent protein mCherry (Figure S2a). SS‐mCherry‐SP_10_ is expected to undertake the following transport route: It is (i) imported into the ER, (ii) then transported to the Golgi through the secretory pathway, (iii) glycosylated in the Golgi via the glycomodules SP_10_, and (iv) finally, secreted to the extracellular medium (Ramos‐Martinez et al., 2017). By transforming the *Chlamydomonas* UVM11 expression strain with the SS‐mCherry‐SP_10_ construct_,_ we generated a secretion strain (Sec) that constitutively secretes SS‐mCherry‐SP_10_ into the culture medium (Figure S2b). Subsequently, we transformed the Sec strain with either the CrSar1‐YFP or the CrSar1DN‐YFP expression vector, thus creating strains that co‐express SS‐mCherry‐SP_10_ and CrSar1‐YFP (hereafter, Sec^Sar1^) or SS‐mCherry‐SP_10_ and CrSar1DN‐YFP (hereafter, Sec^Sar1DN^).

**FIGURE 4:**
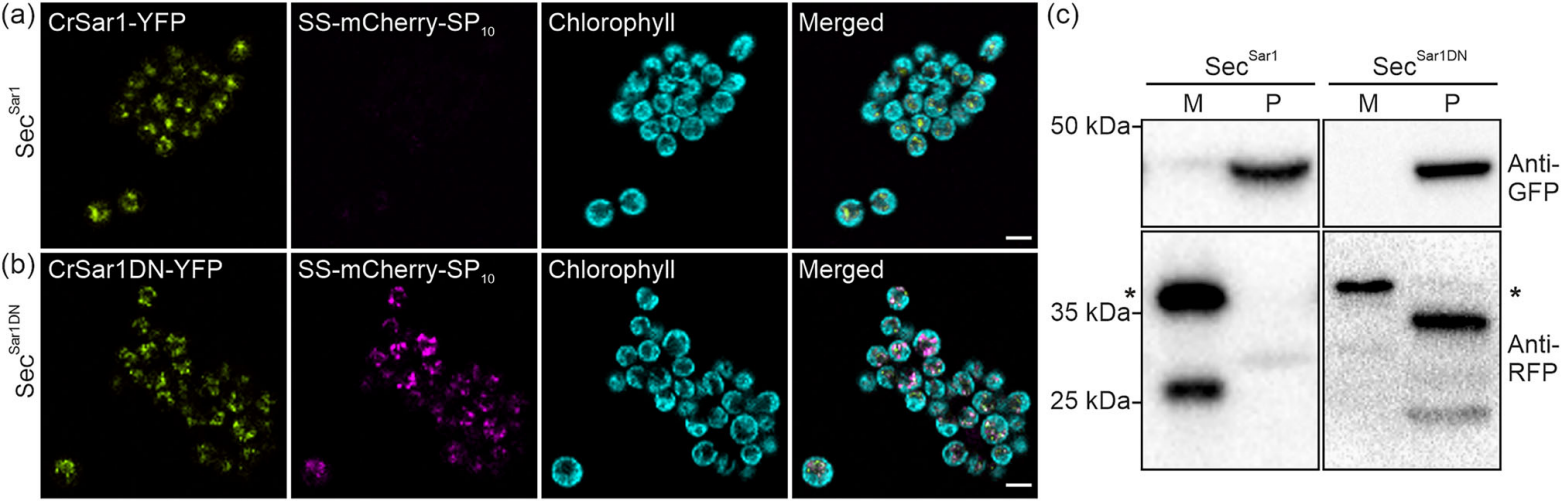
Ectopic expression of the CrSar1DN‐YFP affects protein secretion. (a) Confocal microscopic images of the Sec^Sar1^ strain co‐expressing CrSar1‐YFP (green) and signal sequence (SS)‐mCherry‐SP_10_ (magenta). Chlorophyll fluorescence is shown in cyan. No intracellular mCherry fluorescence signal was observed, suggesting efficient secretion of SS‐mCherry‐SP_10_ to the extracellular medium. Scale bar, 5 μm. (b) Confocal microscopic images of the Sec^Sar1DN^ strain co‐expressing CrSar1DN‐YFP (green) and SS‐mCherry‐SP_10_ (magenta). Chlorophyll fluorescence is shown in cyan. A strong intracellular mCherry fluorescence signal is observed, suggesting that secretion of SS‐mCherry‐SP_10_ is affected by the ectopic expression of CrSar1DN‐YFP. Scale bar, 5 μm. (c) Western blot analysis of protein secretion efficiency in the Sec^Sar1DN^ strain. Secretion efficiency is determined by comparing the relative abundance of the secreted (in the medium fraction, M) and retained (in the cell pellet fraction, P) SS‐mCherry‐SP_10_. Total protein was isolated from both the culture medium and the cell pellet and subjected to separation by sodium dodecyl–sulfate polyacrylamide gel electrophoresis (SDS‐PAGE), followed by immunoblotting using anti‐GFP (for detection of CrSar1‐YFP and CrSar1DN‐YFP) or anti‐RFP (for detection of SS‐mCherry‐SP_10_) antibodies. SS‐mCherry‐SP_10_ was predominately detected in the medium fraction in the Sec^Sar1^ strain while the mCherry signal was detected in both the medium and cell pellet of the Sec^Sar1DN^ strain. Asterisks indicate the fully glycosylated SS‐mCherry‐SP_10_ protein. Bands with lower molecular weights represent non‐glycosylated, partially glycosylated or partially degraded proteins.

We first examined both the Sec^Sar1^ and Sec^Sar1DN^ strains by confocal microscopy. In Sec^Sar1^, we observed only the CrSar1‐YFP signal within the cells (Figure 4a). We were not able to detect any intracellular mCherry signal, consistent with the expectation that the majority of SS‐mCherry‐SP_10_ is secreted into the extracellular medium. By contrast, we detected an intense intracellular mCherry signal in strain Sec^Sar1DN^ (Figure 4b), suggesting a strongly reduced secretion efficiency of SS‐mCherry‐SP_10_. Notably, we observed a partial co‐localization of the YFP and mCherry signals, indicating that SS‐mCherry‐SP_10_ is retained in the same subcellular compartments as CrSar1DN‐YFP.

To verify the inhibitory effect of CrSar1DN‐YFP on protein secretion, we determined the relative abundance of secreted and retained SS‐mCherry‐SP_10_ in both the Sec^Sar1^ and the Sec^Sar1DN^ strains. To this end, we harvested the medium and the cell pellet fractions from the corresponding strains. Western blotting was then performed to determine the extracellular and intracellular abundance of SS‐mCherry‐SP_10_. In Sec^Sar1^, SS‐mCherry‐SP_10_ was detected mostly in the medium fraction, indicating a high secretion efficiency (Figure 4c). In addition to the full‐length protein, a smaller protein was also detected, suggesting instability of the secreted protein in the culture medium, possibly due to attack by extracellular proteases (Hammel et al., 2024). Strikingly, SS‐mCherry‐SP_10_ was readily detected in the cell pellet fraction in Sec^Sar1DN^ (Figure 4c). In addition, multiple bands with smaller sizes than the expected glycosylated SS‐mCherry‐SP_10_ were detected (Figure 4c), suggesting that the processing and glycosylation of the protein cargo is affected in Sec^Sar1DN^.

Taken together, both our microscopic and western blotting data indicate that the ectopic expression of CrSar1DN‐YFP leads to a reduced secretion efficiency of the protein cargo SS‐mCherry‐SP_10._

### 2.5 COPII vesicle budding is not inhibited by the ectopic expression of CrSar1DN‐YFP

Having shown the inhibitory effect of CrSar1DN‐YFP expression on protein secretion, we next wanted to investigate its potential impact on the architecture of the ER‐Golgi interface. Since Sar1 is a key component of the COPII machinery, we were particularly interested in studying the COPII vesicle budding profiles in cells expressing CrSar1DN‐YFP. Therefore, by using cryo‐electron tomography, we examined both the ER‐Golgi architecture and the formation of COPII vesicles at the ultrastructural level.

**FIGURE 5:**
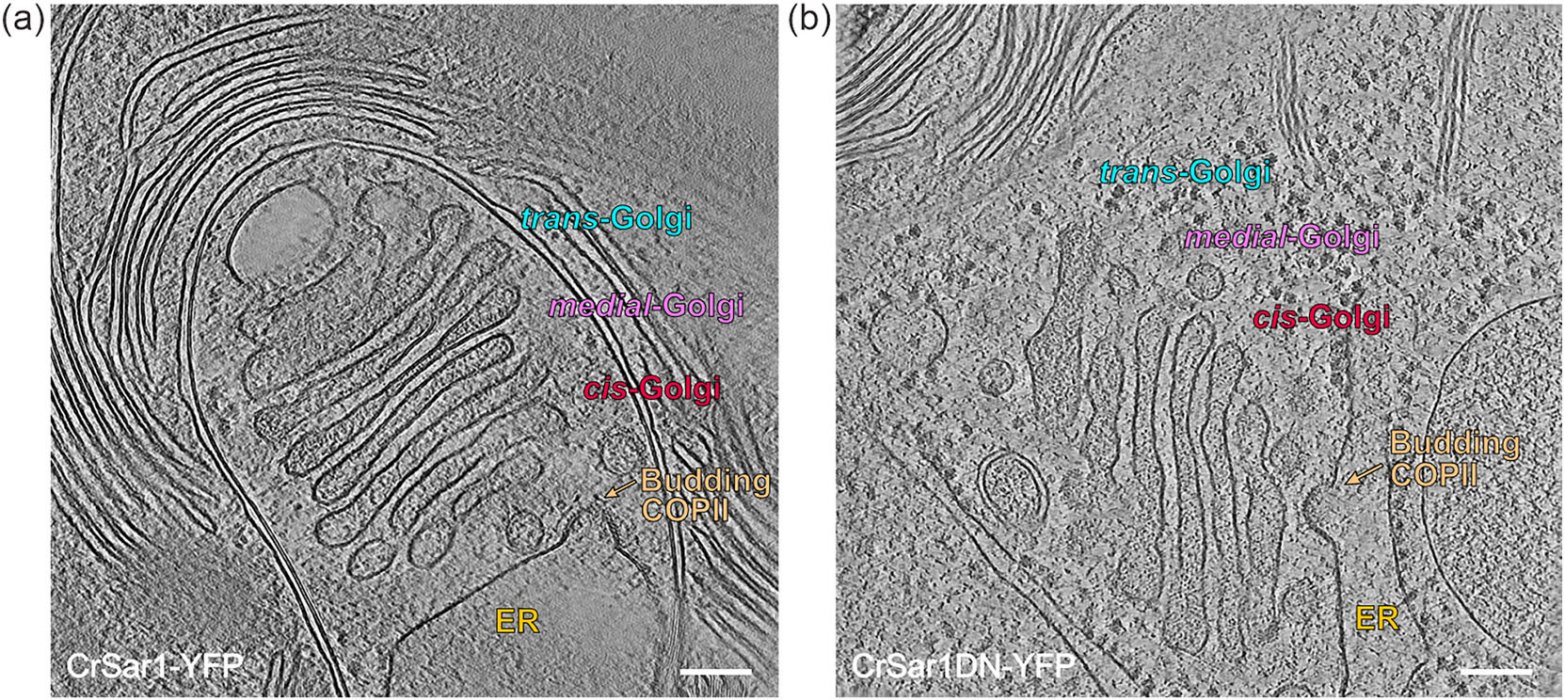
Ectopic expression of CrSar1DN‐YFP does not disrupt the endoplasmic reticulum (ER)‐Golgi interface in *Chlamydomonas*. (a) Slice image of an electron tomogram of cells co‐expressing CrSar1‐YFP and signal sequence (SS)‐mCherry‐SP_10_. The representative image shows the budding of a COPII vesicle (arrow) from the ER. Stacks of the Golgi apparatus in the vicinity of the ER are labeled as *cis*‐, *medial*‐, and *trans*‐Golgi accordingly. Scale bar, 100 nm. For a complete tomogram, see Movie S1. (b) Slice image of an electron tomogram of cells co‐expressing CrSar1DN‐YFP and SS‐mCherry‐SP_10_. The representative image shows the budding of a COPII vesicle (arrow) from the ER. The composition of stacks of the Golgi apparatus in the vicinity of the ER is indicated (as *cis*‐, *medial*‐, and *trans*‐Golgi, respectively). No sign of Golgi collapse was observed in cells expressing CrSar1DN‐YFP, and morphology and architecture of the ER‐Golgi interface are comparable with the Sec^Sar1^ strain. Events of COPII vesicle budding were frequently observed, suggesting that the ectopic expression of CrSar1DN‐YFP does not appreciably inhibit ER‐to‐Golgi protein trafficking. Scale bar, 100 nm. For a complete tomogram, see Movie S2.

To do so, cells of both the CrSar1‐YFP and the CrSar1DN‐YFP strains were plunge frozen, and lamellae were prepared using FIB milling. Tilt‐series were then collected to produce tomograms of the region of interest (see Section 4). Special attention was paid to the ERES regions, where active protein secretion and COPII vesicle budding occur (Budnik & Stephens, 2009; Jensen & Schekman, 2011). In both Sec^Sar1^ and Sec^Sar1DN^ samples, we observed the budding of COPII vesicles from the ER (Figure 5). The overall architecture of the ER‐Golgi interface, the integrity of the ER and the Golgi apparatus, and the morphology and size of COPII vesicles were found to be comparable in the Sec^Sar1^ and Sec^Sar1DN^ samples (Figure 5). Remarkably, we did not observe any pronounced change in the ER‐Golgi interface at the ultrastructural level in cells expressing CrSar1DN‐YFP. Our findings indicate that, in *Chlamydomonas*, the ectopic expression of CrSar1DN‐YFP does not result in the collapse of the ER‐Golgi interface, nor does it cause efficient inhibition of the formation of COPII vesicles.

## 3 DISCUSSION

Sar1, initially identified in yeast, facilitates the formation of COPII vesicles (Nakano & Muramatsu, 1989). Subsequent studies indicated the conserved role of Sar1 in mediating protein export from the ER in various animal and plant species (Brandizzi & Barlowe, 2013; Singh & Jürgens, 2018; Van der Verren & Zanetti, 2023). However, due to the limited knowledge on vesicle formation and trafficking in *Chlamydomonas*, no algal Sar1 homolog had been identified or functionally analyzed.

In this work, we characterized the putative Sar1 homolog in the model green alga *Chlamydomonas* by (i) studying its subcellular localization by expressing a protein fusion with a fluorescent reporter; (ii) creating a dominant‐negative mutant form of CrSar1 using site‐directed mutagenesis; and (iii) analyzing its role in protein secretion using protein secretion assays. Our work shows that CrSar1 possesses many of the defining characteristics of Sar1 that were previously reported in other species.

First, using confocal microscopy, we observed that both CrSar1‐YFP and CrSar1DN‐YFP displayed the cytosolic and punctate localization pattern (Figures 2a and 3a) expected of a protein that functions in association with the ER. Consistent with previous findings from the study of Sar1 in *Arabidopsis* (Hanton et al., 2008, 2009), we found that the CrSar1‐containing and CrSar1DN‐containing punctate structures are indeed closely associated with the ER (Figures 2b and 3b), supporting a potential role of CrSar1 in the ER‐to‐Golgi protein trafficking pathway.

Second, using our established protein secretion assay, we demonstrated the inhibitory role of CrSar1DN on the secretion of the cargo protein SS‐mCherry‐SP_10_ (Figure 4). In *Arabidopsis* and tobacco, the ectopic expression of Sar1DN mutant is known to inhibit ER‐to‐Golgi protein trafficking by titrating COPII components away from vesicle budding sites (DaSilva et al., 2004; Phillipson et al., 2001; Takagi et al., 2020). Notably, we observed a reduced protein secretion efficiency in the Sec^Sar1DN^ strain (Figure 4c), supporting the notion that CrSar1 participates in the early protein secretory pathway.

Using electron microscopy, abundant vesicular transport between the ER and the Golgi apparatus has been reported in *Chlamydomonas* (Donohoe et al., 2013; Hummel et al., 2007). Advanced cryo‐electron tomography has recently made it possible to capture the native structure of both COPII buds and COPII‐coated vesicles at the ER‐Golgi interface (Albert et al., 2020; Bykov et al., 2017), thus providing direct evidence for the occurrence of COPII‐mediated protein trafficking in *Chlamydomonas*. In this work, we have comparatively analyzed the COPII vesicle budding profiles in *Chlamydomonas* Sec^Sar1^ and Sec^Sar1DN^ strains. Consistent with previous findings (DaSilva et al., 2004), we observed Golgi stacks located in the vicinity of the ER. Interestingly, apparent vesicle budding events were seen in both the Sec^Sar1^ and the Sec^Sar1DN^ strains (Figure 5). In contrast to previously studied organisms (Osterrieder et al., 2010; Schäfer et al., 2010; Ward et al., 2001), we did not observe a significant alteration in the architecture of the ER‐Golgi interface nor the COPII vesicle budding profile in the *Chlamydomonas* Sec^Sar1DN^ strain. We, therefore, conclude that the ectopic expression of CrSar1DN‐YFP does not disrupt the COPII machinery, most likely because the endogenous CrSar1 protein still contributes substantially to the formation of COPII vesicles. This observation is in agreement with the findings from the protein secretion assays, which had revealed that the protein cargo SS‐mCherry‐SP_10_ is still detectable in the culture medium of the Sec^Sar1DN^ strain (Figure 4c). Overall, these findings suggest that the COPII‐mediated ER‐to‐Golgi protein trafficking is functional in the Sec^Sar1DN^ strain, albeit operating at reduced efficiency.

Taken together, the high amino acid sequence similarity of CrSar1 with the two *Arabidopsis* Sar1 proteins, our subcellular localization studies, and our protein secretion assays suggest that CrSar1 represents the bona fide algal Sar1 homolog. To gain a comprehensive understanding of the early protein secretory pathway in *Chlamydomonas*, further studies will be required to identify the still missing components of the algal COPII machinery.

## 4 EXPERIMENTAL PROCEDURES

### 4.1 Algal strains and growth conditions

*Chlamydomonas reinhardtii* strain UVM11 (Neupert et al., 2009) was maintained on agar‐solidified Tris‐acetate‐phosphate (TAP) medium at 22–25°C under continuous light (100 μE m^−2^ s^−1^), or in liquid TAP medium on a rotary shaker (120 rpm) at 25°C under continuous light (100 μE m^−2^ s^−1^). Transgenic strains were selected on TAP medium supplemented with paromomycin (15 μg ml^−1^), hygromycin (10 μg ml^−1^), or zeocin (5 μg ml^−1^).

### 4.2 Vector construction

The YFP, mCherry, and mCerulean‐HDEL coding sequences were codon‐optimized for expression in *Chlamydomonas* using the Kazusa CAI table (Nakamura et al., 2000) and chemically synthesized (GeneArt, Regensburg, Germany). Site‐directed mutagenesis was carried out by synthesizing the coding sequence of CrSar1 (Cre11.g468300) with the required mutations in the corresponding nucleotide positions (Figure S1b; GeneArt, Regensburg, Germany). Expression vectors CrSar1‐YFP and CrSar1DN‐YFP were generated by inserting the corresponding transgene into vector pRMB12 (Barahimipour et al., 2015) using the NdeI restriction site. Expression vector SS‐mCherry‐SP_10_ was generated by inserting the corresponding transgene into vector pJR91 (Neupert et al., 2020) using the NdeI restriction site. Expression vector mCerulean‐HDEL was generated by inserting the corresponding transgene into vector pChlamy4 using the EcoRI and PstI restriction sites (GeneArt™ Chlamydomonas Protein Expression Vector). PCR amplification was performed with Phusion High‐Fidelity DNA Polymerase with GC Buffer (Thermo Fisher) following the manufacturer's instructions. All primers used in this study are listed in Table S1. The cloning of all vectors was done in *Escherichia coli* strain DH5α. Large‐scale plasmid preparations were performed using the NucleoBond Xtra Midi kit (Macherey‐Nagel).

### 4.3 Transformation of *Chlamydomonas reinhardtii*

To generate transgenic algal strains, the glass bead‐assisted transformation method was applied (Neupert et al., 2012). Briefly, cultures of the UVM11 strain were grown to the mid‐log phase (i.e., a cell density of 1–2 × 10^6^ cells ml^−1^) in liquid TAP medium. Cells were then harvested by centrifugation at 3000 *g* for 5 min, and the cell pellet was resuspended in TAP medium to reach a cell density of 1 × 10^8^ cells ml^−1^. Of the cell suspension, 300 μl were then mixed with .3‐g glass beads (Sigma‐Aldrich) and 1 μg linearized plasmid DNA of the corresponding transformation vector. The mixture was vortexed for 15 s at maximum speed, followed by spreading on TAP medium plates supplemented with the corresponding antibiotic. The plates were incubated in a growth chamber at 22–25°C under continuous light (100 μE m^−2^ s^−1^) for 2 weeks for selection of transformed colonies.

### 4.4 Fluorescence measurements with the microplate reader

Candidate‐transformed colonies were picked from selection plates and incubated in 100‐μl TAP medium in transparent 96‐well plates (Corning™ Costar™ 96‐well flat‐bottom microplates; Thermo Fisher). Cells were grown to the mid‐log phase in a growth chamber, followed by fluorescence measurement using the CLARIOstar® microplate reader (BMG LABTECH GmbH, Ortenberg, Germany). Excitation at 571 nm (bandwidth 15 nm) and emission at 614 nm (bandwidth 20 nm) were used to measure mCherry fluorescence intensity. The OD_750_ was determined for normalization. Background subtraction and data analysis were performed by the MARS Data Analysis Software (BMG LABTECH GmbH, Ortenberg, Germany).

### 4.5 Protein isolation from cell pellets and the culture medium

Cell cultures of the Sec^Sar1^ and Sec^Sar1DN^ strains were grown to the early logarithmic phase (1 × 10^6^ cells ml^−1^) in liquid TAP medium (10 ml per culture). Cell pellets were harvested by centrifugation at 4000 *g* for 10 min. The supernatant was collected as the culture medium fraction. Aliquots of 7 ml of the culture medium were then transferred into Amicon® Ultra‐15 Centrifugal Filter Units (10 kDa), followed by centrifugation at 4000 *g* for 30 min at 4°C. Culture medium fractions containing secreted cargo proteins were concentrated to a final volume of 250 μl. The cell pellet fractions were resuspended in 200 μl protein extraction buffer (50 mM HEPES/KOH pH 7.5, 10 mM KAc, 5 mM MgAc, 1 mM EDTA, 1% Triton X‐100, 1 mM DTT, 1× protease inhibitor cocktail cOmplete [Roche]). Cell lysis was induced by repeated resuspension (15 times) using a 27G needle with a 1‐ml syringe. The resulting cell lysate was incubated on ice for 30 min, followed by centrifugation at 15,000 *g* for 15 min. The supernatant was recovered as protein extract from the cell pellet fractions. Protein samples from both the culture medium and cell pellet fractions were denatured with SDS‐PAGE sample buffer at 95°C for 5 min.

### 4.6 SDS‐PAGE and western blot analysis

Protein samples were electrophoretically separated in denaturing 12% SDS‐PAA gels, followed by transfer onto polyvinylidene difluoride membranes (Hybond™; GE Healthcare, UK) using standard blotting techniques. The membranes were then incubated with blocking buffer (5% milk powder in 1× TBS‐T) at room temperature for 1 h. After several washing steps with TBS‐T, the membranes were incubated with the primary antibody at 4°C overnight using the following antibody dilutions: anti‐GFP (JL‐8; Clontech, 1:1000), anti‐RFP (6G6, Chromotek, 1:2000). Subsequently, an HRP‐conjugated secondary antibody (mouse, AS111772, 1:25,000, Agrisera) was added and the membranes were incubated at room temperature for 1 h. The ECL Plus™ detection system was used for chemiluminescence signal detection.

### 4.7 Confocal microscopy

Confocal microscopy (TCS SP8, Leica) was performed to examine the subcellular localization of the proteins of interest. YFP fluorescence was detected using an argon laser for excitation at 514 nm, and analyzing emission at 525–555 nm. mCerulean fluorescence was detected using an argon laser for excitation at 458 nm and analyzing emission at 470–510 nm. mCherry fluorescence was detected using an argon laser for excitation at 561 nm, and analyzing emission at 590–630 nm. Chlorophyll fluorescence was detected using an argon laser for excitation at 514 nm, and analyzing emission at 650–700 nm.

### 4.8 Electron tomography

*C. reinhardtii* strains were grown in liquid TAP medium at 22°C to the mid‐log phase (i.e., a cell density of 1–2 × 10^6^ cells ml^−1^) under constant light conditions (~90 μE m^−2^ s^−1^). For vitrification, 4 μl of culture was blotted onto R2/1 carbon‐coated 200‐mesh copper EM grids (Quantifoil Micro Tools) and plunge frozen in liquid ethane/propane mixture using a Vitrobot Mark IV (Thermo Fisher Scientific). EM grids were clipped into Autogrid support rings modified with a cut‐out on one side (Thermo Fischer Scientific) and loaded into Aquilos 2 focused ion beam scanning electron microscopy (FIB‐SEM) instruments (Thermo Fischer Scientific). In the FIB‐SEM, grids were coated with a layer of organometallic platinum to protect the sample surface. Samples were then thinned down to 100–200 nm thick lamellae with a gallium ion beam as previously described (Schaffer et al., 2017). The resulting EM grids with thin lamellae were transferred to a transmission electron microscope for tomographic imaging.

Tomogram acquisition was performed on a 300‐kV Titan Krios G3 microscope (Thermo Fisher Scientific), equipped with a post‐column energy filter (Selectris X, Thermo Fisher Scientific) and a direct detector camera (Falcon4, Thermo Fisher Scientific). Tilt‐series were acquired using Tomo5 (Thermo Fisher Scientific) and a dose‐symmetric tilt scheme (Hagen et al., 2017), with 2° steps totaling 60 tilts per series, with a dose resulting in a total dose per tilt series of approximately 120 e−/Å2. Data were acquired in EER mode at a magnification of 42 k with a calibrated image pixel size of 2.95 Å/pixel. The target defocus of individual tilt‐series ranged from −2.5 to −4 μm with a step of .5 μm. To preprocess and reconstruct the tomograms, TOMOMAN Matlab wrapper scripts (https://github.com/williamnwan/TOMOMAN/; https://doi.org/10.5281/zenodo.4110737) (version 0.6.9) were used. The resulting bin4 tomograms were denoised using Cryo‐CARE (version 0.2.1) (Buchholz et al., 2019) to improve contrast. Denoised tomogram snapshots and movies were captured using the IMOD 3dmod viewer.

## AUTHOR CONTRIBUTIONS

Kin Pan Chung and Ralph Bock designed the experiments. Daniel Frieboese, Kin Pan Chung, and Florent Waltz performed the experiments. All authors interpreted and analyzed the data. Daniel Frieboese and Kin Pan Chung wrote the paper with input from Ralph Bock, Florent Waltz, and Benjamin D. Engel.

## CONFLICT OF INTEREST STATEMENT

The authors declare no conflict of interest.

## ACCESSION NUMBERS

The gene identifiers of the genes mentioned in this study are as follows: *AtSar1a*, At1g09180; *AtSar1b*, At1g56330; *CrSar1*, Cre11.g468300.

## Supporting information


**Table S1.** List of oligonucleotides used in this study.

**Figure S1.** Schematic map of the expression vectors used to express **(a)** CrSar1‐YFP and **(b)** CrSar1DN‐YFP in *Chlamydomonas*. To create a histidine‐to‐leucine substitution at position 74, site‐directed mutagenesis of the corresponding nucleotides was performed. Endogenous *PsaD* sequences were used as expression elements (P: promoter; T: terminator) to drive expression of the transgenes.

**Figure S2.** **(a)** Schematic map of the expression vector SS‐mCherry‐SP_10_. Endogenous *PsaD* sequences were used as expression elements (P: promoter; T: terminator) to drive the expression of the transgene. SS: signal sequence from the metalloprotease gametolysin; SP_10_: synthetic glycomodules. **(b)** mCherry fluorescence intensity measurement in cultures of the UVM11 control strain and the transgenic Sec strain expressing SS‐mCherry‐SP_10_. Fluorescence was measured with a microplate reader, and the fluorescence intensities (arbitrary units) were normalized to the OD_750_ values of the cultures. Fluorescence in the Sec strain is expected to largely come from mCherry secretion into the culture medium (see text for details). The untransformed strain UVM11 was used as a negative control.

**Movie S1.** A complete electron tomogram of cells co‐expressing CrSar1‐YFP and SS‐mCherry‐SP10. Golgi stacks located in the vicinity of the ER were examined (see Figure 5a). Denoised movies were captured using the IMOD 3dmod viewer (see Experimental Procedures).

**Movie S2.** A complete electron tomogram of cells co‐expressing CrSar1DN‐YFP and SS‐mCherry‐SP10. Golgi stacks located in the vicinity of the ER were examined (see Figure 5b). Denoised movies were captured using the IMOD 3dmod viewer (see Experimental Procedures).

## Data Availability

All data generated in this study are included in this published article and the accompanying Supporting information file.
