# Supplementary material for: Identification and characterization of the COPII vesicle‐forming GTPase Sar1 in Chlamydomonas
Source: Plant Direct. 2024 Jun 16;8(6):e614. doi: 10.1002/pld3.614 (PMC11180857; doi:10.1002/pld3.614)
Supplement: Supplementary file 1 — Table S1. List of oligonucleotides used in this study. [file PLD3-8-e614-s004.docx]

**SUPPORTING INFORMATION**

| **Primer** | **DNA target** | **Sequence 5´→ 3´** |
| --- | --- | --- |
| oKPC105 | CrSar1-F | gctactcacaacaagccccaATGTTCCTAGTAAACTGGTTCTATG |
| oKPC106 | CrSar1-R | cagctcctcgcccttgctcaccaTGAATTCCTTGATGTACTGG |
| oDF0001 | mCerulean-HDEL-F | ggagagcaacccgggccccgCGCGTACTGGCGCTCTACTCCTGGTCGCGCTGGCGCTTGCGGGCTGCGCGCAGGCTTGCgugagcaagggcgaggagct |
| oDF0002 | mCerulean-HDEL-R | atttacacggagcggctgcaTTAGAGCTCGTCGTGcttgtacagctcgtccatgcc |
| oKPC169 | SS-mCherry-SP_10_-F | cccactgctactcacaacaagcccatatgatgtcgctggcgacgcgg |
| oKPC170 | SS-mCherry-SP_10_-R | agaattcggtaccttaggggctcggggatggc |

**Table S1.** List of oligonucleotides used in this study.
