# Supplementary figures and images for: Identification and characterization of the COPII vesicle‐forming GTPase Sar1 in Chlamydomonas
Source: Plant Direct. 2024 Jun 16;8(6):e614. doi: 10.1002/pld3.614 (PMC11180857; doi:10.1002/pld3.614)

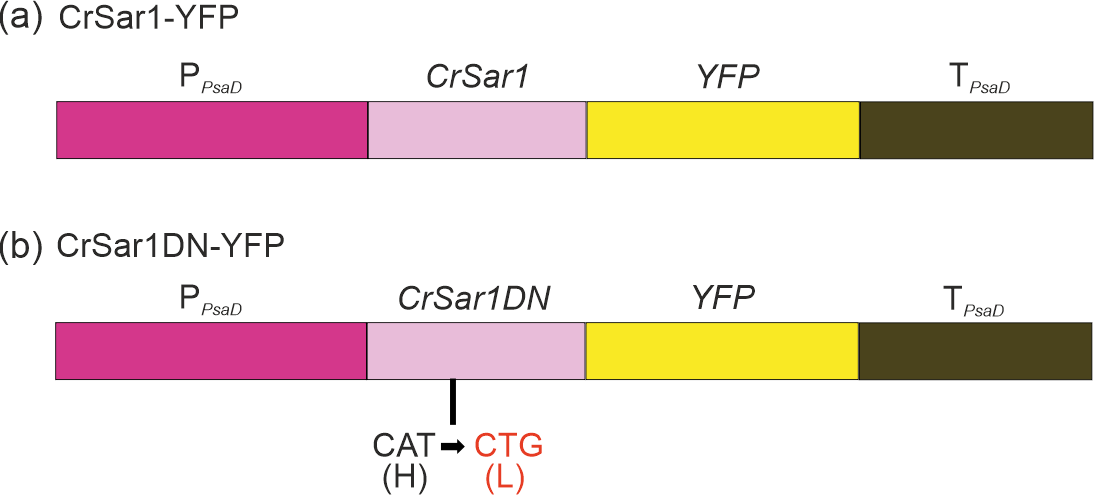

Supplement: Supplementary file 2 — Figure S1. Schematic map of the expression vectors used to express (a) CrSar1‐YFP and (b) CrSar1DN‐YFP in Chlamydomonas. To create a histidine‐to‐leucine substitution at position 74, site‐directed mutagenesis of the corresponding nucleotides was performed. Endogenous PsaD sequences were used as expression elements (P: promoter; T: terminator) to drive expression of the transgenes. [file PLD3-8-e614-s005.tif]

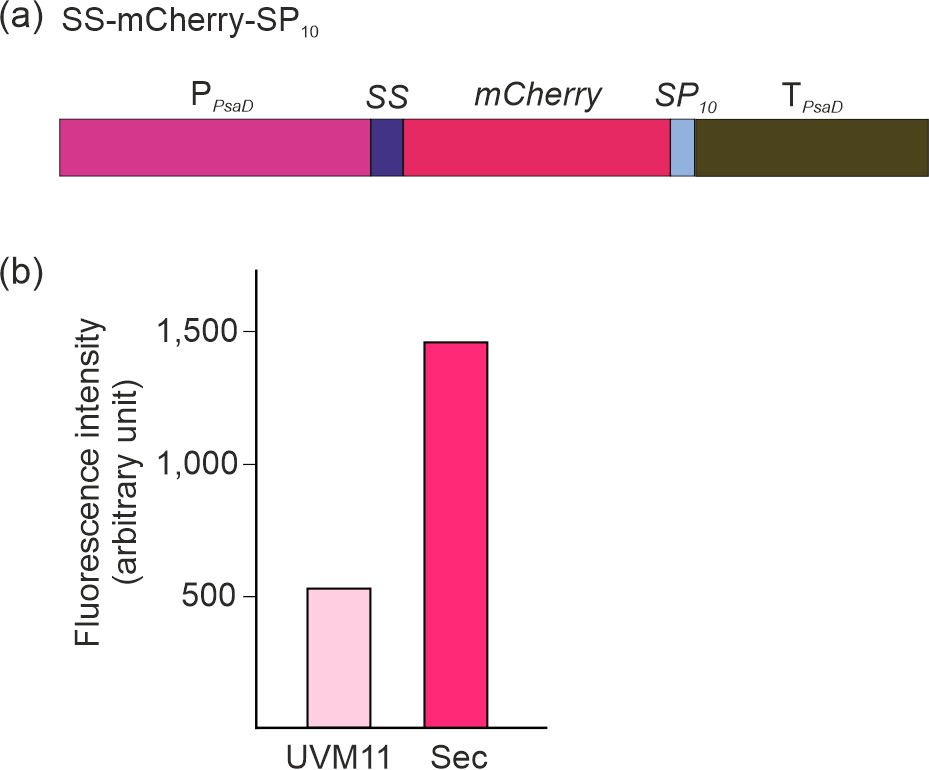

Supplement: Supplementary file 3 — Figure S2. (a) Schematic map of the expression vector SS‐mCherry‐SP10. Endogenous PsaD sequences were used as expression elements (P: promoter; T: terminator) to drive the expression of the transgene. SS: signal sequence from the metalloprotease gametolysin; SP10: synthetic glycomodules. (b) mCherry fluorescence intensity measurement in cultures of the UVM11 control strain and the transgenic Sec strain expressing SS‐mCherry‐SP10. Fluorescence was measured with a microplate reader, and the fluorescence intensities (arbitrary units) were normalized to the OD750 values of the cultures. Fluorescence in the Sec strain is expected to largely come from mCherry secretion into the culture medium (see text for details). The untransformed strain UVM11 was used as a negative control. [file PLD3-8-e614-s002.tif]
